# Endocrine involvement in hepatic glycogen storage diseases: pathophysiology and implications for care

**DOI:** 10.1007/s11154-024-09880-2

**Published:** 2024-04-01

**Authors:** Alessandro Rossi, Chiara Simeoli, Rosario Pivonello, Mariacarolina Salerno, Carmen Rosano, Barbara Brunetti, Pietro Strisciuglio, Annamaria Colao, Giancarlo Parenti, Daniela Melis, Terry G.J. Derks

**Affiliations:** 1 Section of Metabolic Diseases, Beatrix Children’s Hospital, University of Groningen, University Medical Center Groningen, Groningen, The Netherlands; 2 Department of Translational Medicine, Section of Pediatrics, University of Naples “Federico II”, Naples, Italy; 3 Dipartmento di Medicina Clinica e Chirurgia, Sezione di Endocrinologia, Diabetologia ed Andrologia, University of Naples “Federico II”, Naples, Italy; 4 Department of Medicine, Surgery and Dentistry “Scuola Medica Salernitana”, Section of Pediatrics, University of Salerno, Baronissi, Italy; 5 Telethon Institute of Genetics and Medicine, Pozzuoli, Italy

**Keywords:** Glycogen storage disease, Growth hormone, Thyroid, Bone mineral density, Cortisol, Gonads

## Abstract

Hepatic glycogen storage diseases constitute a group of disorders due to defects in the enzymes and transporters involved in glycogen breakdown and synthesis in the liver. Although hypoglycemia and hepatomegaly are the primary manifestations of (most of) hepatic GSDs, involvement of the endocrine system has been reported at multiple levels in individuals with hepatic GSDs. While some endocrine abnormalities (e.g., hypothalamic‑pituitary axis dysfunction in GSD I) can be direct consequence of the genetic defect itself, others (e.g., osteopenia in GSD Ib, insulin-resistance in GSD I and GSD III) may be triggered by the (dietary/medical) treatment. Being aware of the endocrine abnormalities occurring in hepatic GSDs is essential (1) to provide optimized medical care to this group of individuals and (2) to drive research aiming at understanding the disease pathophysiology. In this review, a thorough description of the endocrine manifestations in individuals with hepatic GSDs is presented, including pathophysiological and clinical implications.

## Introduction

Glycogen storage diseases (GSDs) are rare inherited metabolic disorders due to a specific defect in enzymes or transporters involved in glycogen breakdown and synthesis. More than 12 GSD types are recognized causing various symptoms depending on the location of the defect in the glycogen metabolic pathway. Hepatic GSDs (collective estimated incidence of ~ 3:100,000 newborns) are caused by a specific defect in the liver and include GSD type 0a, Ia, Ib, III, IV, VI, IX, and XI. Based on the ability to perform mitochondrial fatty acid oxidation for ketone body production, hepatic GSDs are further classified as ketotic (GSD 0a, GSD III, GSD VI, GSD IX, GSD XI) or non-ketotic (GSD Ia and GSD Ib). The major symptoms and signs in individuals with (most of the) hepatic GSDs are fasting intolerance, hepatomegaly, growth retardation, elevated transaminases and hyperlipidemia [1–4]. Additional findings characterize specific hepatic GSD types (Table 1). Clinical, biochemical and imaging features are traditionally employed for monitoring individuals with hepatic GSDs.

**Table 1:** **Major genetic and clinical features of hepatic glycogen storage diseases (GSDs)**. Fasting intolerance, hepatomegaly (except GSD 0a), growth retardation, hyperlipidemia and elevated transaminases constitute common features of hepatic GSDs and are not shown. Most common findings characterizing each GSD subtype are shown. IBD: inflammatory bowel disease

Type	OMIM	Gene	Locus	Protein defect	Major distinguishing features
0a	240,600	*GYS2*	12p12.2	Glycogen synthase	- Post-prandial hyperglycemia and hyperlactatemia - Absence of hepatomegaly
Ia	232,200	*G6PC1*	17q21.31	Glucose 6-phosphatase-α catalytic subunit	- Elevated lactate and uric acid - Non-/hypo-ketotic hypoglycemia - Renal disease - Liver adenomas may develop
Ib	232,200	*SLC37A4*	11q23.3	Glucose 6-phosphate transporter	- Same as GSD Ia + neutropenia and IBD
IIIa/IIIb	232,400	*AGL*	1p21.2	Glycogen debranching enzyme	- Usually markedly elevated liver transaminases - (Cardio)myopathy (GSDIIIa) - Liver cirrhosis may develop
IV	232,500	*GBE*	3p12.31	Glycogen branching enzyme	- Lack of severe hypoglycemia until end-stage liver disease - Liver cirrhosis may present early in infancy
VI IXa IXb IXc	232,700 306,000 261,750 613,027	*PYGL* *PHKA2* *PHKB* *PHKC*	14q22.1 Xp22.13 16q12.2 16q11.2	Liver glycogen phosphorylase Phosphorylase kinase α subunit Phosphorylase kinase β subunit Phosphorylase kinase γ subunit	- Hypoglycemia is usually mild
XI^1^	227,810	*SLC2A2*	3q26.2	GLUT2	- Post-prandial hyperglycemia - Renal tubular disease (Fanconi syndrome)

^1^ also known as Fanconi-Bickel syndrome

Dietary management including frequent feedings, regular uncooked corn starch (UCCS) intake, gastric-drip feeding is the cornerstone of the treatment for hepatic GSDs [5, 6]. Pharmacological therapy (e.g., lipid-lowering drugs, granulocyte colony-stimulating factor, ACE-inhibitors) can correct secondary metabolic disturbances and/or prevent/delay disease complications. Additional treatment options (e.g. radiofrequency ablation, liver transplantation) can be considered when previous options are not sufficiently effective [1].

**Fig. 1:**
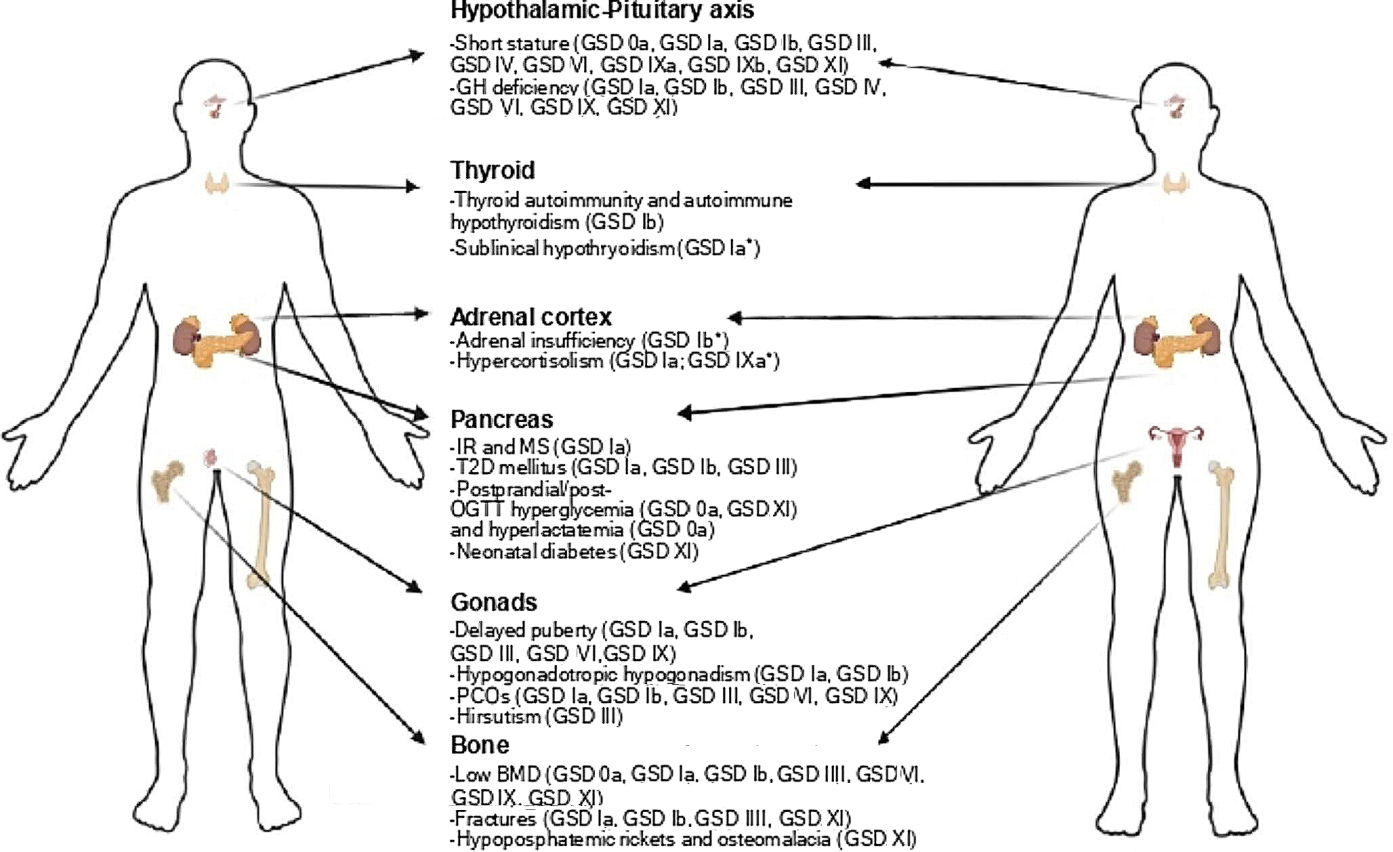
Endocrine manifestations of hepatic glycogen storage diseases (GSDs). For each endocrine component, clinical and biochemical features are presented. Specific GSD subtypes are indicated in brackets. BMD: bone mineral density; GH: growth hormone; IR: insulin-resistance; MS: metabolic syndrome; OGTT: oral glucose tolerance test; PCOs: Polycystic ovaries; T2D: type 2 diabetes *only 1 individual reported

Despite the treatment, individuals with hepatic GSDs can experience metabolic decompensation [7] and develop a number of (long-term) complications, including liver adenomas and renal failure [8, 9]. Among those, disruption of the endocrine system has been extensively reported at multiple levels in hepatic GSDs [10–14]. An overview on the involvement of the different endocrine axes in individuals with hepatic GSDs is provided, including pathophysiological and clinical implications. A summary of major endocrine manifestations observed in hepatic GSDs is presented in Fig. 1; Table 2.

**Table 2:** **Endocrine manifestations in specific hepatic glycogen storage disease (GSD) types**. For each (group of) GSD type clinical manifestations and related treatments are displayed according to the different endocrine components presented in the main text. Treatment options are displayed in italics when there is limited supporting evidence

GSD Type	ENDOCRINE MANIFESTATIONS											
	Hypothalamic-Pituitary axis^1^		Thyroid Gland		Bone		Pancreas		Adrenal cortex		Gonads^1^	
	Clinical features	Treatment	Clinical features	Treatment	Clinical features	Treatment^7^	Clinical features	Treatment	Clinical features	Treatment	Clinical features	Treatment
**0a**	FTT Short stature	Diet^3^	-	-	Low BMD	Diet Mineral^6^ Vitamin^6^ *BP*	Postprandial hyperglycemia and hyperlactatemia	Diet	-	-	-	-
**Ia**	FTT Short stature GH deficiency	Diet^3^ rhGH^4^ *OLT*	Subclinical hypothyroidism*	*L-T4*	Low BMD Bone fractures	Diet Calcium Vitamin^6^ *BP*	IR MS T2D mellitus Diabetic ketoacidosis* Hyperglucagonemia	Diet *α-glucosidase* *inhibitors* *Insulin* *SGLT2-inhibitors*	Hypercortisolism	Diet	Delayed puberty Hypogonadotropic hypogonadism PCOs Irregular menses Menorrhagia	Diet *Testosterone* ^*9*^ Avoid estroprogestative pills
**Ib**	FTT Short stature GH deficiency APAs	Diet^3^ rhGH^4^ *OLT*	Thyroid autoimmunity Hypothyroidism^5^	*L-T4*	Low BMD Bone fractures	Diet Calcium Vitamin^6^ *BP*	T2D mellitus	Diet *Insulin*	Hypocortisolism	Diet	Delayed puberty Hypogonadotropic hypogonadism PCOs Irregular menses Menorrhagia	Diet *Testosterone* ^*9*^ Avoid estroprogestative pills
**IIIa/IIIb**	FTT Short stature GH deficiency	Diet^3^ rhGH^4^	-	-	Low BMD Bone fractures	Diet Mineral^6^ Vitamin^6^ *BP*	T2D mellitus	Diet *α-glucosidase* *inhibitors* *Insulin*	-	-	Delayed puberty PCOs Hirsutism	Diet
**IV**	FTT Short stature GH deficiency	Diet^3^ rhGH^4^ *OLT*	-	-	-	-	-	-	-	-	-	-
**VI** **IXa** **IXb** **IXc**	FTT Short stature GH deficiency^2^	Diet^3^ rhGH^4^	-	-	Low BMD	Diet Mineral^6^ Vitamin^6^ *BP*	-	-	Hypercortisolism^2^*	Diet	Delayed puberty PCOs	Diet
**XI**	FTT Short stature GH deficiency	Diet^3^ Electrolytes Calcitriol Bicarbonate L-carnitine	-	-	Low BMD Bone fractures Hypophosphatemic rickets Osteomalacia	Diet Bicarbonate Phosphate Calcitriol Vitamin^6^ *BP*	Postprandial/post-OGTT hyperglycaemia Neonatal diabetes^8^	Diet SGLT-2 inhibitors*	-	-	Delayed puberty	Diet

^1^ In all hepatic GSD types catch-up growth and recovering delayed puberty may be observed when proper (dietary) treatment is initiated ^2^ Reported in GSD IXa ^3^ Dietary treatment includes (a combination of) frequent feedings, uncooked cornstarch and gastric-drip feeding ^4^ Only in case of proven GH deficiency and after nutritional therapy has been optimized ^5^ Overt or subclinical ^6^ As per individual need ^7^ Physical activity is encouraged in all GSD types ^8^ Transient or permanent ^9^ Only after nutritional therapy has been optimized * Only one patient reported. APAs: anti-pituitary antibodies; BMD: bone mineral density; BP: bisphosphonates; FTT: failure to thrive; IR: insulin resistance; MS: metabolic syndrome; OGTT: oral glucose tolerance test; OLT: Orthotopic liver transplantation; PCOs: polycystic ovaries; T2D: type 2 diabetes

### Hypothalamic-pituitary axis

Failure to thrive and short stature are frequent findings in children with hepatic GSDs [8, 15]. Their prevalence spans from 10% in GSD 0a [16] and GSD III [17] to 17% in GSD IXb [18], 27% in GSD Ia [19], 30% in GSD IXa [18], 38% in GSD Ib [19], 52% in GSD VI [20], 57% in GSD IV patients [21] and up to 90% of GSD XI individuals [22–25]. Initial growth retardation together with a late growth spurt are common features. A subsequent catch-up growth is usually observed when proper (dietary) treatment is initiated [26]. However, some adult individuals can still experience short stature despite adequate treatment compliance [15, 27].

The underlying mechanism is only partly understood but it is assumed to result from the combination of chronic hypoglycemia, lactic/keto-acidosis, and abnormal hormonal response, including growth hormone (GH)-insulin-like growth factor (IGF-1) axis alteration [14, 28–30]. The extent of glucose metabolism derangement may explain why spontaneous catch-up growth can be observed even in untreated children with ketotic GSD types, while it only occurs in children with GSD I upon dietary treatment initiation [10, 31]. In GSD I disruption of gluconeogenesis results in the accumulation of lactate with no (or little) increase in circulating ketones. In ketotic GSDs gluconeogenesis is intact (thus preventing hyperlactatemia) and circulating ketone levels are increased; in these GSD subtypes ketones serve as an alternative energy substrate thus sparing glucose [17]. Nonetheless, chronic ketosis as well as amino acid depletion from gluconeogenesis could worsen growth pattern even in ketotic GSD types [32, 33]. Indeed, chronic hypoglycemia and metabolic (keto)acidosis can decrease the amplitude and frequency of GH pulses in experimental metabolic acidosis [34]. In humans, chronic metabolic acidosis is associated with decreased serum IGF-1 concentration and is related to a resistance to the hepatocellular action of GH [35]. Blunted GH response can also result from elevated circulating free fatty acids (FFA) [36], which is commonly observed in individuals with GSD I who display suboptimal glucose control [10, 37].

Besides the above-mentioned “functional” GH deficiency, growth retardation may also arise from impaired GH secretion. This “structural” GH deficiency likely results from (combination of) deranged glucose metabolism in the pituitary gland or disease-induced pituitary autoimmunity [38]. Indeed, GH deficiency has been variously reported in individuals with inherited metabolic disorders in which phosphorylated, simple carbohydrates accumulate, either due to the primary metabolic defect, or associated with dietary (over)treatment. Examples include GSD Ia [29, 39, 40], GSD Ib [38, 41, 42], GSD III [43], GSD IXa [44], GSD XI [45], phosphoglucomutase 1 deficiency (also called PGM1-CDG) [46] and Fructose-1,6-Bisphosphatase (FBPase) deficiency [47]. Interestingly, similar to the latter two disorders, the authors have observed that individuals with hepatic GSDs may display abnormal carbohydrate-deficient transferrin testing. The question is to what extent an overarching pathophysiology mechanism may play a (partial) role in the development of GH deficiency. Particularly, in GSD Ib higher prevalence of anti-pituitary antibodies has been detected, possibly resulting from immune cell dysfunction observed in this disorder [38].

Additional endocrine imbalance may also contribute to growth failure as an adaptation to glucose metabolism derangement. Indeed, individuals with GSD I and poor growth have been shown to exhibit low serum insulin concentration and higher mean 24-hour plasma cortisol levels as compared to better grown individuals [14]. These findings suggest that chronic hypoglycemia may affect multiple endocrine axes in hepatic GSDs.

Both renal tubular dysfunction and impaired liver glucose homeostasis may play a role in the development of growth failure in GSD XI [11, 22–24, 48]. Whether failure to thrive is the result of intestinal malabsorption by impaired glucose transport across enterocytes and/or impairment of monosaccharide transport in renal tubular cells together with hyperaminoaciduria is not fully understood [24]. Furthermore, glucose is an important energy source for the metabolism and growth of chondrocytes. Perturbations of glucose metabolism affect chondrocyte maturation and cartilage matrix production, suggesting a key role for glucose metabolism during endochondral ossification [49]. Interestingly, immunoreactivity of GLUT2 has been detected in the hypertrophic zone of the epiphyseal growth plate in growing rats [50]. However, the impact of pathogenetic *GLUT2* variants on cartilage development is yet to be elucidated.

In the medical care, besides traditional biomedical biomarkers routinely employed for monitoring individuals with hepatic GSDs, the following anthropometric parameters should be regularly assessed: (i) height; (ii) weight; (iii) weight/height ratio or body mass index depending on age; v) head circumference in children [5, 51, 52]. Changes in growth trends may reflect either poor metabolic control/overtreatment or disease progression prompting (dietary) treatment adjustments [5, 33, 48, 51, 52]. Close clinical and biochemical monitoring is particularly relevant during periods of rapid growth [33]. Regular evaluation of glucose homeostasis is recommended in patients with all hepatic GSD types. Assessment of baseline IGF-1 levels as well as provocative GH testing should be considered in individuals with unexplained failure to thrive and short stature. Additionally, pituitary autoimmunity should be investigated in individuals with GSD Ib displaying a growth defect [38]. Urine electrolytes and plasma carnitine should be monitored in individuals with GSD XI [48].

Overall, adequate metabolic control together with optimization of dietary treatment are paramount to possibly ensure regular growth in individuals with hepatic GSDs [4, 5, 8, 14, 17, 19, 23, 31, 33, 51–55]. Catch-up growth as well as recovery of height potential has been reported in various hepatic GSD types when proper treatment is initiated [11, 31, 48, 16, 55–59]. The goal of dietary treatment is to maintain normal blood glucose and ketone concentrations by providing appropriate amounts of complex carbohydrates. This can be achieved by (a combination of) frequent feedings, UCCS and (nocturnal) gastric-drip feeding [5, 33, 48]. Children with ketotic GSDs should also be started on a high protein diet to sustain gluconeogenesis [51, 60]. A high-lipid diet may be of benefit in individuals with GSD III [61]. Electrolytes, calcitriol, bicarbonate, and L-carnitine should be supplemented in individuals with GSD XI [48].

Growth hormone (rhGH) therapy is not routinely indicated in hepatic GSDs unless GH deficiency has been proven and only after nutritional therapy has been optimized. Although rhGH can ensure proper growth in GSD I [40, 41], GSD III [43] and GSD XI [45], this treatment is concerning due to the potential increased risk of developing liver adenomas [4, 5, 33, 51, 52]. The possible mechanism remains unresolved but is likely related to promotion of tumor cell migration and/or energy rewiring in metabolically injured hepatocytes [27]. Furthermore, rhGH therapy may exacerbate (extreme) lipid [5, 43, 44] and ketone [4, 33] elevation. Hence, treatment with rhGH should be coupled with strict patient monitoring and use of lipid-lowering agents if needed [41]. Although liver transplantation is a potential treatment option in children with GSD I [6, 62, 64] and GSD IV [52] displaying growth failure, the results after liver transplantation reported in the medical literature point in variable directions [63, 64].

### Thyroid gland

Thyroid involvement has only been reported in GSD I. Thyroid autoimmunity with overt or subclinical hypothyroidism has been described in individuals with GSD Ib [13, 65]. Besides primary thyroid damage, enhanced thyrotropin response to thyrotropin releasing hormone has been observed in both GSD Ia and GSD Ib [13], suggesting that concomitant damage at the level of the hypothalamus or pituitary gland may exist in GSD I. More recently, subclinical hypothyroidism has been reported in one individual with GSD Ia [66].

Although the mechanism underlying the development of hypothyroidism in GSD Ib is not fully understood, it appears to be related to the increased risk of autoimmunity with abnormal T-cell function and neutropenia observed in this disorder [67]. As for GSD Ia, whether the occurrence of hypothyroidism is the result of enzymatic defect per se, chronic liver disease or incidental association remains unclear. Interestingly, decreased hepatic triglycerides content was found in *G6pc*^−/−^-deficient mice treated with VK2809 (a liver-specific thyroid hormone receptor β-agonist [68]), suggesting that thyroid dysfunction may concur to the progression of liver disease in GSD I. Future studies elucidating the pathophysiology of hypothyroidism in GSD I are warranted.

Based on the above-mentioned findings, early diagnosis and treatment of thyroid disorders are paramount to improve the prognosis of individuals with GSD I [8]. This is particularly relevant as the risk of autoimmunity increases as patients progress into adulthood [69]. Annual monitoring of TSH and fT4 levels and thyroid hormone supplementation in case of hypothyroidism are recommended in individuals with GSD I [1]. When a pregnancy is possible, pre-conceptional fT4 and TSH should be assessed, taking into account the known influence of even subclinical hypothyroidism on early fetal brain development and long-term cognitive function [69, 70]. Overt hypothyroidism is associated with increased rates of spontaneous abortion, premature delivery and/or low birth weight, fetal distress in labor, and likely gestational hypertension, emphasizing the importance of thyroid balance before and during pregnancy [71].

### Bone

Low bone mineral density (BMD) and higher risk of developing osteopenia (i.e. Z-score < 1.0) [72], osteoporosis (i.e. Z-score < 2.0) [73] and fractures have been reported in both children and adult individuals with GSD type 0a [16], I [1], III [2], VI [4, 33], IX [33, 74] and XI [23]. Fractures have been observed in up to 17% of individuals with hepatic GSDs [75, 76]. Hypoposphatemic rickets are commonly found in untreated patients with GSD XI between 3 and 10 months of age [77]. Despite showing normal circulating calcium levels, studies performed in individuals with GSD I and GSD III suggest the presence of both reduced bone deposition and increased bone remodeling [12, 78].

The pathophysiology of bone involvement in hepatic GSDs appears to be multifactorial, stemming from the combination of abnormal metabolic environment (e.g. elevated lactate, elevated ketones), nutritional deficiency, and possibly hormonal imbalance as well as altered muscle physiology [76, 78]. Both ketosis and hyperlactatemia exert a detrimental effect on bone [79]. Hyperlipidemia is also known to blunt bone anabolism [80]. Indeed, a correlation between BMD and circulating lactate and/or triglycerides has been reported in patients with GSD I and GSD III [12, 75, 78]. Notably, optimized metabolic control is associated with improved BMD [12]. Dietary treatment may also contribute to low BMD. Reduced dietary calcium intake [81, 82] as well as decreased circulating 25-OH vitamin D levels have been observed [18, 75, 83]. Historical studies reported that higher protein intake worsened bone health [84, 85]. Yet, growing literature suggests that high-protein diet may not have adverse effects on BMD [86–88]. Endocrine imbalance may also contribute to decreased BMD. Decreased circulating parathyroid hormone (PTH), calcitonin and osteocalcin have been reported in individuals with GSD I and GSD III [12, 78]. Similarly, GH deficiency [27, 29, 38, 40, 43], hypogonadism [89] as well as chronically low insulin [14, 78] and/or elevated cortisol levels [90] may play a role in the development of osteopenia/osteoporosis. Furthermore, failure of glucose supply to the exercising muscle together with impairment of the (endocrine regulation of the) muscle-bone unit appear to be major contributors to low BMD in GSD III [78]. In these disorder IGF-1/Insulin-like growth factor-binding protein 3 (IGFBP3) ratio appears to be a reliable biomarker of reduced BMD [78]. A correlation between BMD and age at start and duration of granulocyte colony-stimulating factor (G-CSF) treatment was found in individuals with GSD Ib [12].

The pathophysiology of bone demineralization is different in GSD XI, where patients are more prone to develop hypocalcemia and hypercalciuria, hyperphosphaturia with or without hypophosphatemic rickets in early childhood, osteoporosis and osteomalacia [23, 24, 91–94]. Ketosis, chronic metabolic acidosis with or without diarrhea, proximal renal tubular dysfunction, aberrant interplay among PTH, vitamin D and FGF23 are possible contributing factors [77, 91].

In the medical care, measurement of BMD together with circulating 25-OH vitamin D and dietary calcium and vitamin D intake is recommended at the diagnosis in all individuals with hepatic GSDs [5]. Subsequent evaluations are usually performed every 3–5 years or as clinically indicated [1, 2]. Assessment of circulating 25-OH vitamin D levels is indicated annually, or more frequently as needed [1, 2, 77]. Regular assessment of alkaline phosphatase, total calcium, PTH, calciuria, and phosphaturia may be useful for treatment monitoring [95]. Additional endocrine work-up should be performed if clinically indicated. Dual-emission X-ray absorptiometry (DXA) is the gold standard technique for BMD assessment being usually performed at the hip. L1-L4 vetrebrae should be considered in growing children as the hip is not a reliable site. Being a safe, inexpensive and nonradiation method for bone density assessment, Quantitative Ultrasound (QUS) has been proposed as an alternative method for low BMD diagnosis and follow-up in children [12, 78]. Signs of hypophosphatemic rickets should be regularly checked and promptly identified in all children, particularly those with GSD XI including: (i) swelling of joints; (ii) bowing of the legs; (iii) pathological fractures; (iv) teeth problems with a susceptibility to develop severe caries [24].

Good metabolic control, including adequate dietary compliance has been shown to improve BMD in individuals with hepatic GSDs [2, 5, 96]. Given the restricted dietary regimen, supplementation with calcium and/or multivitamins is strongly recommended to prevent osteopenia/osteoporosis in GSD I [5, 97]. Recommendations for vitamin and mineral supplementation in other GSD types should be based on individual patient diet and nutrient needs [33, 51]. Calcium supplementation should be tailored based on renal function, given the risk of kidney stone formation [98]. If necessary, supplementation of vitamin D can be prescribed to ameliorate bone mineralization [2]. 1,25-dihydroxy vitamin D is indicated in individuals with GSD XI [91, 92]. Particularly in individuals with GSD III, physical activity should be encouraged in order to protect the bone [51, 81].

In individuals with GSD Ib under G-CSF treatment, the risk of osteopenia/osteoporosis should be carefully monitored. The demonstration of an association between osteopenia and G-CSF treatment suggests using the minimally effective G-CSF dose. This association also adds to the growing evidence pointing in favour of the use of empagliflozin as a first line treatment for neutropenia/neutrophil dysfunction in individuals with GSD Ib [99, 100]. In individuals with GSD XI sodium bicarbonate and phosphate supplementation are additionally indicated to prevent bone loss and hypophosphatemic rickets [24, 91] and to enhance growth velocity [22, 24, 77]. Alkali supplementation (e.g. in form of Shohl’s solution or bicarbonate solution) can be considered to minimize the hypercalciuria [24, 77]. Phosphate should be supplemented as oral Joulie’s solution [92].

In principle, low BMD can lead to (recurrent) fractures in both children and adults [101]. In the general population bisphosphonates (BP) are indicated in children with osteoporosis and pathological fractures or vertebral fractures regardless of Z-score [102]. However, an “acute phase reaction” (e.g. fever, malaise, back pain, body pains, nausea, and vomiting) following initial dose of BP is commonly observed. Moreover, hypocalcemia can occur as a short-term side effect related to BP therapy [103]. Hence, the role of BP in asymptomatic individuals with hepatic GSDs and decreased BMD is still controversial and currently there is no recommendation for their use [78, 81]. However, there is evidence of improvement of BMD in single individuals with hepatic GSDs treated with BP [75, 76]. When considering whether to start a patient with hepatic GSD on BP therapy, the following factors should be considered: (i) individual’s age (currently there is no evidence-based data to support their use in children); (ii) evidence of increased bone destruction [75, 76].

### Pancreas

Increased prevalence of insulin resistance (IR) and metabolic syndrome (MS) has been reported in individuals with GSD Ia [11, 104–107]. Type 2 diabetes (T2D) mellitus has been reported in individuals with GSD Ia [108–112] and GSD Ib [113, 114] and found in up to 9% of individuals with GSD III [17, 115–118]. However, diabetic ketoacidosis secondary to T2D has been observed in only one young girl with GSD Ia [112]. Historical studies have also reported increased glucagon levels in GSD I [60, 119–121]. Postprandial hyperglycemia and hyperlactatemia are common findings in individuals with GSD 0a [16]. A combination of chronic glycosuria and postprandial hyperglycemia together with post-oral glucose tolerance test (OGTT) hyperglycemia can be detected in GSD XI especially in the younger patients [22, 77, 122]. Individuals with GSD XI may also develop transient or permanent neonatal diabetes [123–127].

IR may result from the combination of several factors. Dietary overtreatment (i.e. high carbohydrate/UCCS intake) may lead to hyperglycemia, hyperinsulinaemia, obesity and rebound hypoglycaemia [6, 82, 128]. In GSD Ia IR may also develop as a consequence of the G6Pase-α deficiency *per se.* G6P excess in endoplasmatic reticulum, may upregulate the activity of 11β-hydroxysteroid dehydrogenase type 1 (11βHSD1) which results in increased conversion of inactive cortisone in active cortisol [90, 106]. Increased circulating cortisol levels may lead to metabolic syndrome [129]. Furthermore, mitochondrial dysfunction as well as accumulation of lipid metabolism by-products may contribute to IR in GSD Ia [107, 130, 131]. Downregulation of the glucose receptor on the ß-cell membrane (GLUT2) as an adaptation to hypoglycemic events may also accur leading to blunted insulin secretion in response to transient elevations of blood glucose [114]. Although pathophysiology of T2D in GSD I and GSD III is strictly correlated to IR [108–110, 112, 114, 116–118, 132], additional determinants may also contribute including (i) injured fatty liver [109, 110]; (ii) pancreatic islet β-cell insufficiency as a results of recurrent pancreatitis [109, 114] and (iii) liver cirrhosis in GSD III [133]. Notably, T2D has been observed in two siblings with GSD Ib even following liver transplantation, supporting this hypothesis [113]. Increased glucagon levels have been found in individuals with GSD I [119–121, 134] but not in GSD III [134]. This finding has been associated to hyperlactatemia [119, 120] and may reflect preserved gluconeogenic amino acids availability in GSD III [134].

Postprandial hyperglycemia in GSD 0a results from the inability to store glucose as glycogen in the liver (due to glycogen synthase defect) rather than impaired insulin secretion [16, 135]. Pathogenesis of postprandial/post-OGTT hyperglycaemia in GSD XI has been recently reviewed [136]. While fasting hypoglycemia is due to impaired glucose transport out of the hepatocytes, postprandial hyperglycemia likely results from hypoinsulinemia secondary to altered sensitivity of pancreatic beta cells to glucose [122, 123]. As such, insulin response is decreased but not absent in these individuals [122]. Postprandial/post-OGTT hyperglycaemia has been especially observed in younger patients. Likely glucose transport improves in older patients through GLUT2-independent mechanisms (e.g. GLUT1, GLUT3) [122, 123, 137]. This may also explain transient or permanent neonatal diabetes which has been rarely reported in GSD XI [123–127]. However it may be possible that some cases with transient neonatal diabetes remain undiagnosed [137]. More recently overexpression of circulating miRNAs correlated with type 1 diabetes mellitus has been found in one individual with GSD XI [136].

In the medical care, besides traditional biomedical monitoring biomarkers the following parameters should be regularly evaluated: (i) height; (ii) weight; (iii) weight/height ratio or body mass index depending on age; ; (iv) circulating insulin levels. Evaluation of circulating cortisol and ACTH may be performed in individuals with GSD Ia who display IR despite dietary optimization [90]. For early detection and management of glucose intolerance an OGTT may be considered [118]. Yet, OGTT remains contraindicated in women with hepatic GSDs due to the increased risk of hypoglycaemia [138].

Reaching appropriate diagnosis in a patient with hyperglycemia and glycosuria is essential. GSD 0a rather than diabetes mellitus should be considered in the differential diagnosis of postprandial hyperglycaemia when polyuria and polydipsia are absent [139]. Given the association of postprandial hyperglycaemia alternating with ketotic hypoglycaemia, GSD 0a and GSD XI could be reciprocally misdiagnosed. However, postprandial hyperlactatemia is observed in GSD 0a but not GSD XI [139].

Prevention of IR is paramount in hepatic GSDs. Due to the risk of iatrogenic hyperinsulinism, regular diet assessment is recommended and excess feeding/UCCS intake should be avoided [5]. In this respect, a metabolic dietitian should work closely with the patients to refine the dietary plan. Continuous glucose monitoring (CGM) appears particularly helpful in optimizing dietary treatment. Indeed, CGM allows unveiling both hypoglycemia and hyperglycemia which may be missed by traditional capillary glucose monitoring [108–110, 140–146]. Overall, adequate metabolic control together with regular reassessment of dietary plan aim to ensure optimal outcome in individuals with hepatic GSDs [6, 8, 17, 128]. Providing appropriate amounts of UCCS and complex carbohydrates is particularly relevant as glucose requirements decrease with age in individuals with hepatic GSDs [128]. IR may worsen (cardio)myopathy in GSD III by depleting energy substrates (i.e. fatty acids and ketone bodies) and promoting glycogen storage [147]. Hence, dietary treatment paradigm is being revised for this disorder with accumulating evidence indicating a benefit of a high-fat low-carbohydrate diet [61, 148]. Whether simple sugars (e.g. fructose, sucrose and galactose) should be life-long restricted in GSD I to avoid rapid insulin secretion [5, 6, 149] is still controversial.

The optimal pharmacological treatment for IR in individuals with hepatic GSDs is as yet undefined. Hypoglycemic agents (e.g. insulin and insulin secretagogues) are not routinely indicated as they can precipitate hypoglycemia [16, 77, 150]. Nonetheless, single patients successfully treated with voglibose [117], acarbose [110], insulin [112, 115, 116, 118], canagliflozin [110] and luseogliflozin [132] have been reported. The use of the sodium-glucose co-transporter 2 (SGLT2) inhibitors (e.g. empagliflozin, dapagliflozin) is widely spreading for treatment of neutropenia-related symptoms in GSD Ib [99, 100, 151–155]. Additionally, one adult patient with GSD XI treated with dapagliflozin displayed reduced glycogen content in shed urinary cells and improved serum potassium and phosphate concentrations [156]. Notably, side effects of SGLT2 inhibitors include elevated lactate and ketoacidosis, especially under stress conditions (e.g. intercurrent infections and major surgery) [157] prompting careful monitoring.

### Adrenal cortex

Limited data on adrenal cortex hormones are available in hepatic GSDs. Two historical studies revealed an inverse correlation between plasma cortisol levels and growth parameters [10, 14] in GSD I. More recently, systematic adrenal cortex assessment has been performed in individuals with GSD I [90]. During normoglycemia, increased baseline and ACTH-stimulated serum cortisol levels were found in individuals with GSD Ia, while those with GSD Ib exhibited decreased baseline serum cortisol levels [90]. Furthermore increased plasma corticosterone and epinephrine levels have been found in fasted GSD Ia mouse model [158]. High midnight serum cortisol concentrations have been detected in one untreated boy with GSD IXa who presented with Cushing-like appearance [159].

The mechanism leading to imbalanced cortisol levels in GSD I is yet to be elucidated. Disrupted cortisol metabolism may result from the G6P modulation of the ER-bound enzyme 11β-HSD1, which activates cortisone to cortisol [160]. 11β-HSD1 is typically expressed in glucocorticoid receptor-rich tissues, such as the liver (where G6Pase-α is also expressed), adipose tissue, lung and brain [161]. The otherwise preserved adrenal cortex function suggests that disrupted cortisol metabolism might be secondary to local deregulation rather than hypothalamic-pituitary-adrenal axis dysfunction. Increased cortisol regeneration may represent a potential mechanism to divert lipid excess in GSDIa [90]. Indeed, administration of glucocorticoid receptor- antagonist mifepristone, has been shown to prevent Very-low-density lipoprotein (VLDL) accumulation in *g6pc*^*−/−*^ mouse [158]. In addition, recurrent hypoglycemia may likely result in a “stress-induced Cushing syndrome” [159]. These observations warrant mechanistic studies, especially in light of the ongoing, experimental AAV8-mediated gene therapy treatment, which is currently in phase 3, in which temporary treatment with corticosteroids is indicated (NCT05139316). Currently, there are no recommendations on monitoring of adrenal cortex function in patients with hepatic GSDs. Hence, the need for such assessments remains on an individual basis. Evaluation of adrenal cortex function in individuals with hepatic GSDs displaying poor metabolic control is worthy. Reaching good metabolic control may contribute to reverse hypercortisolism [159]. Future studies investigating the effects of agents modulating glucocorticoid metabolism are warranted.

### Gonads

Gonadal involvement has been documented in GSD type I [89, 105, 162, 163], III [105, 133, 164], VI [11], IX [11] and XI [23, 24], including delayed puberty, hypogonadotropic hypogonadism and Polycystic ovaries (PCOs).

Functional delayed puberty is a recognized feature of untreated chronic diseases [165]. Consistently, delayed puberty has been reported in GSD type I, III, VI and IX and XI, likely due to suboptimal metabolic control secondary to poor dietary compliance [4, 5, 17, 22, 23, 31, 33, 51]. A relationship between dietary treatment and pubertal development has been described in several individuals [31, 55, 166, 167]. Failure to thrive together with delayed puberty has been reported in a boy with GSD Ia following voluntary discontinuation of UCCS [166]. Catch-up growth and pubertal development together with normalization of blood testosterone levels were noticed in a 16-year-old boy diagnosed with GSD Ia following institution of dietary treatment [167]. Although dietary treatment plays a role in growth and sexual development, the mechanism underlying delayed puberty in hepatic GSDs is still not fully understood. At least in theory, delayed puberty may also result from hormonal imbalance observed in hepatic GSDs, involving circulating insulin and cortisol levels [10]. A correlation between serum insulin and cortisol levels and growth has been demonstrated in individuals with GSD I [14]. Whether such hormonal imbalance results from the enzyme defect per se or is secondary to (poor) dietary treatment remains to be elucidated.

Hypogonadotropic hypogonadism has been described in males with GSD I, showing low luteinizing hormone (LH) and follicular stimulating hormone (FSH), and correspondingly low total testosterone levels [89]. All individuals displayed recurrent hypoglycaemia and elevated lactate levels, suggesting a possible relation with suboptimal metabolic control [89]. Indeed, chronic recurrent elevations of cortisol in response to hypoglycemia may lead to suppression of gonadotropin-releasing hormone (GnRH), LH and FSH release [168].

PCOs are more commonly observed in women with GSD I, in whom they have been documented as early as 5 years of age [5, 11, 105, 163]. Less frequently PCOs are reported in other GSD types [11, 17, 33, 51, 105, 133]. Although PCOs are main features of Polycystic ovary syndrome together with hyperandogenism and irregular mensens, hyperandrogenism is an infrequent finding in hepatic GSDs [11, 105] being hirsutism reported in some women with GSDIII [17, 51, 133]. Conversely, irregular menses and menorrhagia are commonly associated with PCOs in GSD type I [162, 163] and less frequently in other GSD subtypes [17, 51, 133, 162].The mechanism underlying the development of PCOs in hepatic GSDs remains incompletely understood. Lower serum sex hormone-binding globulin (SHBG) levels have been reported in individuals with GSD Ia displaying an inverse association with intrahepatic lipid content, thus supporting a connection between metabolic (im)balance and circulating sex hormone levels [169]. Hyperinsulinism is commonly observed in suboptimally treated individuals [105] indicating a potential role for the diet in the development of PCOs. Whether good dietary compliance is sufficient to ensure adequate ovarian function in hepatic GSDs is, however, unclear [163]. Interestingly, PCOs are also observed in patients with Cushing’s syndrome [170]. Therefore, imbalanced cortisol levels may also concur to PCOs development in hepatic GSDs [90]. Future studies elucidating the underling mechanisms of PCOs are warranted.

In the medical care, besides traditional biomedical monitoring biomarkers, the following assessments should be regularly performed: (i) pubertal development in children and adolescents; (ii) frequency and regularity of menses, uncovering possible menorrhagia or irregular menstrual bleeding; (iii) signs of hyperinsulinism and/or hypercortisolism (e.g. increased weight and/or waist circumference and altered systolic and/or diastolic blood pressure); (iv) signs of hyperandrogenism, (e.g. acne, alopecia, and hirsutism) [171]. Incorporating clinical and/or biochemical screening of the hypothalamic-pituitary–gonadal axis is be important in the management of hypogonadism in males with hepatic GSDs [89]. Women with hepatic GSDs should be made aware of the increasing risk of severe hypoglycaemia in the premenstrual and luteal phase [172]. Pelvic ultrasonography should be performed regularly in women with hepatic GSDs to document PCOs [133].

Overall, adequate metabolic control is paramount to possibly ensure regular gonadal function in hepatic GSDs [4, 5, 17, 22, 23, 31, 33, 51, 163]. Indeed, puberty can be near normal with appropriate metabolic control [8, 31]. Sex hormone replacement is the most commonly employed treatment for delayed puberty in the general population [173]. Yet, estrogen therapy is not routinely indicated in women with GSD I as estrogens contribute to development of liver neoplasms [174]. Although testosterone replacement therapy allows development or maintenance of secondary sexual characteristics in males with hypogonadotropic hypogonadism [89], patients with hepatic GSDs should be carefully monitored due to the stimulation of hepatocyte proliferation by androgens [89, 174]. When indicated, transdermal estrogens are preferred over oral preparations due to hepatic first-pass metabolism [173]. Estrogen therapy in postmenopausal women may increase the risk of venous thromboembolism and stroke whereas reduces the risk of breast cancer and bone fractures [175]. Conversely, testosterone replacement therapy has not been associated with a significant elevation in the rates of venous thromboembolism and cardiovascular events [176, 177]. As oral testosterone may increase cardiovascular risk [178], intramuscular or transdermal administration should be preferred [178].

Classical combined estrogen-progestogen contraception as well as oral estrogens should be avoided in young women with hepatic GSDs, given the high risk of adenomas onset [5, 51, 179]. Progestin-only contraceptives may be considered [5, 51]. However, clinicians should be aware of the risk for reduced BMD, which needs to be monitored [5, 51]. The use of an intrauterine device should be avoided in GSD Ib, given the high risk of infection [5].

Successful pregnancies have been reported in women with GSD 0a [180], GSD I [151, 181–185], GSD III [186–189], GSD VI [190] and GSD XI [191] either spontaneously or after fertility treatment [190]. Male individuals with GSD I, GSD III [56] and GSD XI [192] who became fathers have been reported.

Pregnancy should be planned ahead of time and a careful management by a multidisciplinary health care team is required [184]. Good metabolic control together with close blood glucose monitoring and regular adjustments in diet and UCCS dosing are required before conception and throughout pregnancy to ensure successful outcomes [138]. Indeed, maternal hypoglycemia may be associated with intrauterine growth restriction and low birth weight [189]. Increasing protein intake may be necessary to provide an alternate source for glucose via gluconeogenesis in ketotic GSD types [33, 51]. Given the association between high estrogen state during pregnancy and adenoma onset [179], women with GSD I should be made aware of the increased risk of enlargement and rupture of adenomas [163].

## Conclusions

Hepatic GSDs are complex disorders, requiring a highly specialized multidisciplinary team to achieve treatment goals [2, 5, 6, 20, 33, 51, 52]. Their multisystem involvement raises significant organizational, logistic, and financial obstacles for affected families and healthcare providers. The potentially life-threatening nature of hepatic GSDs symptoms and high variability in patients’ phenotypes, treatment interventions and outcomes emphasize the need and urgency for improved monitoring options.

The progress in dietary treatment as well as the availability of appropriate tools to manage acute metabolic decompensation [7] has shifted the clinical focus from “mortality” to “morbidity”. As a result, a number of long-term complications have emerged, including those affecting the endocrine system. In this review we provided a comprehensive summary of endocrine involvement in hepatic GSDs. Being aware of the endocrine manifestations of hepatic GSDs would have two main benefits: (i) optimized disease management, improving patient outcome and possibly allowing standardization of clinical care; (ii) earlier identification of hepatic GSDs in individuals displaying milder phenotypes; this appears particularly relevant as such individuals may first come to the (pediatric) endocrinologist attention without having been referred by a metabolic specialist.

Disruption of the endocrine system may occur at multiple levels in hepatic GSDs resulting in various (serious) clinical conditions. These include short stature, hypothyroidism, osteopenia/osteoporosis, IR and PCOs, among others (Fig. 1; Table 2). Currently available evidence argues in favour of regular screening for endocrine function in individuals diagnosed with hepatic GSDs in order to start prompt treatment. Appropriate treatment stems from the exact knowledge of the mechanisms underlying each endocrine condition. Many endocrine manifestations (e.g. failure to thrive, osteopenia/osteoporosis, IR, delayed puberty) share a multifactorial pathogenesis, thus complicating the use of targeted approaches. In these cases, current management strategy relies on optimization of (dietary) treatment for hepatic GSDs. In specific cases (e.g. short stature, hypogonadism) a distinct hormone deficiency can be identified, supporting hormone replacement therapy. At least in theory additional mechanisms may concur to endocrine dysfunction in hepatic GSDs, including relationship between energy production and hormone synthesis, effect of toxic metabolite accumulation or hormone/receptor glycosylation. Indeed, depletion of gluconeogenic amino acid precursors (which are employed for endogenous glucose production) may contribute to growth failure in ketotic GSDs [134, 149, 193]. Nonetheless, (glycogen-derived) UDP-glucose is required for glycosylation of glycoprotein hormones such as TSH, FSH and LH [194]. Elucidating such mechanisms may improve current knowledge of disease pathophysiology and potentially develop novel monitoring and treatment tools.

Several innovative treatment strategies are currently being investigated for hepatic GSDs, including gene replacement/base editing (NCT05139316, NCT05095727) [195], anaplerotic therapy (NCT03665636) and drug repurposing (NCT04138251; NCT05960617; NCT04986735). The aim of such approaches is to either restore energy balance or prevent the accumulation of a toxic metabolite. Growing evidence supports the efficacy of these treatments on “classical” disease manifestations (e.g. fasting intolerance, neutrophil dysfunction) [152, 196]. Whether these approaches are also effective on (long-term) endocrine manifestations in hepatic GSDs is yet to be determined.

Delivering standardized high-quality healthcare to patients worldwide is among the top priorities for hepatic GSDs [142, 197]. To this aim, current evidence on endocrine involvement in hepatic GSDs as well as management suggestions were presented in this review. This work also underlines the compelling need to strengthen multistakeholder collaborative networks including both metabolic and endocrine experts to optimize patient care.

## Data Availability

No datasets were generated or analysed during the current study.

## Abbreviations

GSDs: Glycogen storage diseases
BMD: Low bone mineral density
BP: Bisphosphonates
CGM: Continuous glucose monitoring
DXA: Dual-emission X-ray absorptiometry
FBPase: Fructose-1,6-Bisphosphatase
FFA: Free fatty acids
FSH: Follicular stimulating hormone
G-CSF: Granulocyte colony-stimulating factor
GH: Growth hormone
GLUT2: Glucose receptor on the ß-cell membrane
GnRH: Gonadotropin-releasing hormone
IGF-1: Insulin-like growth factor
IR: Insulin resistance
LH: Luteinizing hormone
OGTT: Post-oral glucose tolerance test
PCOs: Polycystic ovaries
PGM1-CDG: Phosphoglucomutase 1 deficiency
PTH: Parathyroid hormone
QUS: Quantitative Ultrasound
rhGH: Recombinant human growth hormone
SGLT2: Sodium-glucose co-transporter 2
SHBG: Serum sex hormone-binding globulin
T2D: Type 2 diabetes
UCCS: Uncooked corn starch
VLDL: Very-low-density lipoprotein
11βHSD1: 11β-hydroxysteroid dehydrogenase type 1
